# Atorvastatin as an Antihypertensive Agent: A Pilot Study

**DOI:** 10.7759/cureus.49532

**Published:** 2023-11-27

**Authors:** Niaz Ali, Muhammad Faheem, Himayat Ullah, Hosam Shabana, Arafat Kassem, Mahmoud O Ahmed, Essam Elmahdi

**Affiliations:** 1 Pharmacology, College of Medicine, Shaqra University, Shaqra, SAU; 2 Pharmacology, Ayub Medical College, Abbottabad, PAK; 3 Medicine, College of Medicine, Shaqra University, Shaqra, SAU; 4 Internal Medicine, Faculty of Medicine, Al-Azhar University, Cairo, EGY; 5 Internal Medicine, Faculty of Medicine, Mansoura University, Al Mansoura, EGY

**Keywords:** atorvastatin, anti-hypertensive, calcium channel blockers, statins, hypercholesterolemia, hypertension

## Abstract

Objective

Hypertension (HTN) is among the most common causes of chronic disease burden, along with dyslipidemia. It is a prominent risk factor for cardiovascular and cerebrovascular morbidity and mortality. More often than not, HTN coexists with dyslipidemia. This study aimed to see the antihypertensive effect of statins (atorvastatin), as certain animal models have shown that statins have a voltage-gated calcium channel-blocking effect.

Material and methods

This was a randomized controlled trial done at the Ayub Hospital Complex in Abbottabad, Pakistan. After ethical approval, 120 patients with newly diagnosed hypertension belonging to either gender and aged 35 and above were enrolled in the trial. They were randomly divided into two groups, with each group comprising 60 patients. One group was administered amlodipine 5 mg per oral (PO) once a day, while the other group was given 5 mg of amlodipine PO plus 10 mg of atorvastatin PO. The patients were examined on a follow-up visit 14 days later, and blood pressure was recorded as per protocols.

Results

A total of 120 newly diagnosed patients were studied in this trial. The mean age was 51.07 years, with a standard deviation of ±6.15 years and a range of 41-60 years. There were 64 (53.3%) males and 56 (46.7%) females in the study. The mean systolic blood pressures (SBPs) and diastolic blood pressures (DBPs) in Group 2 (amlodipine 5 mg + atorvastatin 10 mg) were significantly lower than the patients in Group 1 (only amlodipine 5 mg) in the follow-up visit, which was 14 days after starting the medication (p≤0.05).

Conclusion

The addition of a lipid-lowering drug to an antihypertensive regimen results in a better lowering of blood pressure in hypertensive individuals.

## Introduction

Hypertension (HTN) is defined by the World Health Organization (WHO) as “a condition in which the blood vessels have persistently raised pressure, putting them under increased stress” [1]. It is usually asymptomatic, but chronically raised blood pressure (BP) damages the vascular endothelium, leading to end organ damage in the form of ischemic heart disease, stroke (both ischemic and hemorrhagic), renal vascular disease, peripheral vascular disease, and retinopathy [2-5]. Uncontrolled BP is one of the most common causes of death worldwide [6]. The majority of the international guidelines define hypertension as clinic systolic blood pressure (SBP) of ≥140 mmHg or diastolic blood pressure (DBP) of ≥90 mmHg on several occasions [7-9]. The European Society for Hypertension (ESH) and the National Institute of Clinical Excellence (NICE) guidelines have a more acceptable and practical approach to ambulatory blood pressure monitoring (ABPM) or home blood pressure monitoring (HBPM), defining hypertension as SBP ≥135 mmHg and DBP ≥85 mmHg [10,11]. This technique minimizes the risk of misdiagnosing white coat hypertension as HTN [11,12].

Currently, various regimens are used for the treatment of HTN. These include angiotensin-converting enzyme inhibitors (ACEIs) and angiotensin receptor blockers (ARBs), calcium channel blockers (CCBs), diuretics, beta receptor blockers, alpha receptor blockers, etc. Statins, which are widely used as lipid-lowering drugs, have been shown to lower both SBP and DBP in meta-analyses, though they are not used as an antihypertensive agent [13, 14]. Statins, discovered in 1976, inhibit 3-hydroxy-3-methylglutarylcoenzymeA (HMG-CoA) reductase, which is the rate-limiting step in cholesterol synthesis [15]. It leads to the inhibition of mevalonate synthesis, which is the cholesterol precursor. Statins have been shown to enhance nitric oxide bioavailability and thus vascular compliance in some experimental studies [13]. This mechanism is most probably associated with the BP-lowering effect of statins. It has been shown in certain reports that calcium channels are being regulated by statins, resulting in their synergistic effect [16].

Some studies have shown that statins along with Ca++ channel blockers are more effective in lowering BP than calcium channel blockers alone [17]. Certain animal models have already shown that statins have voltage-gated calcium channel-blocking activity [18]. For this purpose, we designed a clinical trial to see the antihypertensive effect of statin (atorvastatin) in hypertensive patients.

## Materials and methods

Design

This study was conducted in the Ayub Hospital Complex in Abbottabad, Pakistan, after ethical committee approval (approval number: DIR/KMU-EB/EA/000560, dated January 28, 2019). The trial was registered at ClinicalTrials.gov with ID NCT05679102. The sample size was calculated by a standardized sample size calculator (Raosoft sample size calculator, Raosoft Inc., Seattle, WA), keeping the confidence interval at 95% and the margin of error at 5%, using the annual incidence of newly diagnosed HTN as 8.4%, according to the studies [19]. The sample size came out to be 118 patients. A total of 120 patients of both genders, aged 35 years and older, were recruited for this study. The sampling technique used was non-probability convenience sampling. The whole process was explained to the patients, and informed consent was obtained from the participants.

Inclusion and exclusion criteria

The inclusion criteria for the study were all newly diagnosed hypertensive patients aged 35 years and older with normal lipid profiles. Patients with a history of dyslipidemia, diabetes mellitus, chronic kidney disease, and ischemic heart disease were excluded from the study. Other possible confounders like BMI, lifestyle, physical activity, and smoking were addressed by taking the sample from the same population with no significant difference between these variables and distributing the number of smokers almost equally in both groups (Group 1 had eight smokers and Group 2 had nine smokers). For the diagnosis of HTN, office BP on three separate occasions over two weeks was taken, and the patients with BP ≥140/90 mmHg were labeled as hypertensive. The baseline BP of patients was checked in sitting and standing positions using the same mercury sphygmomanometer for all the participants. They were randomly divided into two groups, 1 and 2. Patients in Group 1 were given amlodipine 5 mg per oral (PO) for their HTN, while those in Group 2 were given 5 mg of amlodipine PO plus 10 mg of atorvastatin PO. Blood pressure was recorded on the respective steady-state concentration of the drugs after 14 days of treatment initiation.

Statistical analysis

The data collected were analyzed by MS Excel (Microsoft Corporation, Redmond, WA) and IBM SPSS software version 22 (IBM Corporation, Armonk, NY). Numerical variables were described as mean ± SD, while categorical variables were described as frequencies and percentages; an ANOVA at a 95% confidence level (p ≤0.05) was used to test the significance. The results were expressed as tables and graphs.

## Results

A total of 120 hypertensive patients, including 64 males (53.3%) and 56 females (46.7%), were enrolled in this trial.

The mean (± SD) age of these study participants was 51.07 (± 6.15) years. The pre-treatment mean (± SD) SBPs and DBPs of all the patients (both Groups 1 and 2) were 169.95 (± 6.02) mmHg and 98.63 (± 4.28) mmHg, respectively. The number of males and females in the amlodipine group (Group 1) was similar, i.e., 30 (50%), while males (n=34; 56.7%) outnumbered females (n=26; 43.3%) in the amlodipine plus atorvastatin group (Group 2). Table 1 depicts the gender distribution of both groups.

**Table 1:** Gender distribution of participants among Groups 1 and 2

Parameter	Male		Female		Total	
	Frequency	Percentage	Frequency	Percentage	Frequency	Percentage
Group 1	30	50.0	30	50.0	60	50
Group 2	34	57.0	26	43.0	60	50
Total	64	53.3	56	46.7	120	100

The post-treatment mean (± SD) SBPs and DBPs of patients included in both Groups 1 and 2 were 150.74 (±6.48) mmHg and 83.64 (±5.41) mmHg, respectively. Table 2 summarizes these values.

**Table 2:** Cumulative statistics of both Groups 1 and 2 BP: blood pressure

Parameter	Minimum	Maximum	Mean	Standard deviation
Age (years)	41	60	51.07	6.148
Pre-intervention				
Systolic BP (mmHg)	160	180	169.95	6.021
Diastolic BP (mmHg)	90	105	98.63	4.282
Post-intervention				
Systolic BP (mmHg)	136	165	150.74	6.482
Diastolic BP (mmHg)	75	95	83.64	5.409

Patients in the amlodipine group were slightly older than those receiving both amlodipine and atorvastatin. The mean SBP in these patients was slightly higher than in Group 2, while the DBP was slightly lower than in Group 2. The final SBPs and DBPs in Group 1 were higher than the BP recorded in Group 2 patients. The age, baseline, and final SBPs and DBPs of patients in Group 1 and Group 2 are summarized in Tables 3-4, respectively.

**Table 3:** Group 1 statistics: amlodipine only BP: blood pressure

Parameter	Minimum	Maximum	Mean	Standard deviation
Age (years)	41	60	51.18	6.492
Pre-intervention				
Systolic BP (mmHg)	160	180	168.38	5.188
Diastolic BP (mmHg)	90	105	98.27	4.524
Post-intervention				
Systolic BP (mmHg)	147	165	153.45	6.029
Diastolic BP (mmHg)	75	95	87.47	4.890

**Table 4:** Group 2 statistics: amlodipine + atorvastatin BP: blood pressure

Parameter	Minimum	Maximum	Mean	Standard deviation
Age (years)	41	60	50.95	5.835
Pre-intervention				
Systolic BP (mmHg)	160	180	171.52	6.419
Diastolic BP (mmHg)	90	105	99.00	4.030
Post-intervention				
Systolic BP (mmHg)	136	155	147.50	5.513
Diastolic BP (mmHg)	75	85	79.82	2.311

**Figure 1:**
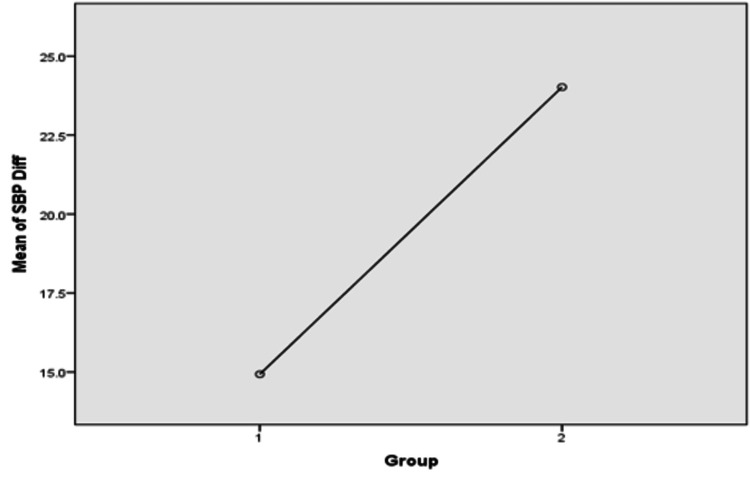
An ANOVA plot comparing the differences (diff) between the means of pre-treatment and post-treatment systolic blood pressure (SBP) in Groups 1 and 2

**Figure 2:**
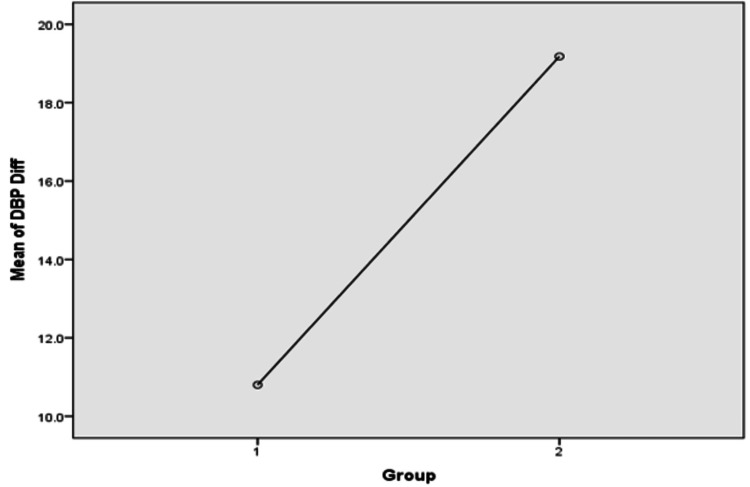
An ANOVA plot comparing the differences (diff) between the means of pre-treatment and post-treatment diastolic blood pressure (DBP) in Groups 1 and 2

A one-way ANOVA was applied to compare the difference between mean SPBs and DBPs before and after antihypertensive treatment in the two groups, and the result of the ANOVA showed that there was a statistically significant decrease in the mean SPBs and DBPs of Group 2 as compared to that of Group 1 (p <0.01) (Table 5, Figures 1, 2)

**Table 5:** One-way ANOVA for the difference between means of pre-treatment and post-treatment systolic blood pressure (SBP) and diastolic blood pressure (DBP) of the two groups (p-value=.001).

Parameter		Sum of squares	df	Mean square	F	p-value
SBP difference	Between groups	2478.843	1	2478.843	1249.791	.000
	Within groups	234.042	118	1.983		
	Total	2712.885	119			
DBP difference	Between groups	2106.732	1	2106.732	979.350	.000
	Within groups	253.836	118	2.151		
	Total	2360.568	119			

## Discussion

In this study, we found that the difference between the pre-treatment and post-treatment SBP of Group 1 was 14.93 mmHg, while that of Group 2 was 24.02 mmHg. Similarly, the difference between the pre-treatment and post-treatment DBP of Group 1 was 10.8 mmHg, while that of Group 2 was 19.18 mmHg. This clearly showed that the co-administration of amlodipine and atorvastatin has significantly better control of BP as compared to amlodipine alone. Chang et al., in their study, concluded a similar inference [17]. In their study, they aimed to measure the synergistic effect of atorvastatin and amlodipine on BP control. Their study also reported the possible synergistic effect of these two drugs on left ventricular remodeling. The difference between this study and ours is that they conducted it on pre-existing hypertensive patients and patients with deranged lipid profiles (dyslipidemia). Another large randomized control trial called the Brisighella Heart Study done by Borghi et al. also obtained similar results in lowering BP [20]. This study showed that of 1,356 patients using lipid-lowering drugs for dyslipidemia, those using statins for dyslipidemia had better BP control than those using other drugs. Also, this BP-lowering effect was more pronounced in patients with SBP greater than or equal to 140 mmHg.

Since these studies favor our findings, yet these studies were done on hypertensive patients with dyslipidemia; one can argue that lowering serum lipid levels can indirectly improve BP control. This argument was also favored by some studies, and one of the studies categorically inferred that the addition of statin to antihypertensive medications only lowers BP in hypertensive patients who have poor BP control and also higher blood cholesterol levels [21]. This statement was somewhat contradictory to ours, that adding a statin to antihypertensive drugs, especially calcium channel blockers, will show better control of BP even in patients with a normal lipid profile. To establish the fact that statins lower BP even in patients without dyslipidemia, we selected patients who had normal serum lipid profiles. A similar effect was reported by Koh et al. in their study, in which they stated that statins lower the BP in hypertensive patients even if they have normal blood cholesterol levels. However, they added that this BP-lowering effect is only limited to those with elevated BP and has no effect on those with normal BP [22]. In one of their 14-week multicenter trials, Blank R et al. found that amlodipine plus atorvastatin combination therapy was found to be successful in lowering BP and a deranged lipid profile in more than 57% of study participants, showing synergistic effects of the two drugs [23]. However, the issue with these studies previously done on the effect of statins on BP control was that the population used to be chronically hypertensive, already using other approved antihypertensive therapies. So, one can object since these patients are already on antihypertensive therapy, which may add to the effects of the trailed drugs. To remove this confounder, we selected newly diagnosed hypertensive patients who had not previously taken any antihypertensive medications. We also kept the duration of follow-up shorter (14 days) so that the antihypertensive effect of atorvastatin, if any, can be established well before the proper lipid-lowering effect of atorvastatin has taken place.

Though we did not study the synergistic effect of amlodipine plus atorvastatin on lowering BP, studies are concluding the synergy between the two drugs [24,25]. One of the drawbacks of this study was the lack of long-term follow-up to see whether this antihypertensive effect of atorvastatin is constant or just a transient effect. We also did not check the dose relationship of atorvastatin with BP lowering. Apart from this, the sample size was also an issue, but the reason for this is the difficulty in following up with these newly diagnosed hypertensive patients, who, without any hypertensive complications, usually do not visit hospitals often in our setup. This study can be a pilot study for future research. One of the limitations of the study was the small sample size, so large population-based studies are required to determine the magnitude of the synergistic effect in population-based samples. However, it can safely be recommended that the serum lipid profile of newly diagnosed hypertensive patients be checked, and the patients should be offered statin as a lipid-lowering therapy in conjunction with antihypertensive therapy wherever suitable.

## Conclusions

Combining hydroxymethylglutaryl-CoA (HMG-CoA) reductase inhibitors (simply known as statins) with antihypertensive drugs results in better BP lowering compared to monotherapy in hypertension. This effect of statins on lowering BP is due to vasodilation, as shown in certain animal models, and is independent of their lipid-lowering effect. This vasodilatory effect can play a synergistic role with the calcium channel blockers as they are also vasodilators, but this requires large-scale studies. The long-term effects of such action will strongly relate to a reduction in coronary artery disease risk, as per previously published research on the risk reduction of coronary artery diseases. Large-scale studies are required to approve the combination of a statin with an antihypertensive, preferably a calcium channel blocker, for better BP control than that antihypertensive alone.
